# Role of NaCl and Glutamine on Biofilm Production from *Pseudomonas aeruginosa*

**DOI:** 10.3390/microorganisms13092198

**Published:** 2025-09-19

**Authors:** Laura Maria De Plano, Antonella Iaconis, Salvatore Papasergi, Francesco Mediati, Daniele Caruso, Salvatore Pietro Paolo Guglielmino, Domenico Franco

**Affiliations:** 1 Department of Chemical, Biological, Pharmaceutical and Environmental Sciences (ChiBioFarAm), University of Messina, Viale F. Stagno d’Alcontres 31, 98166 Messina, Italy; ldeplano@unime.it (L.M.D.P.); antonella.iaconis@unime.it (A.I.); francesco.mediati@studenti.unime.it (F.M.); sguglielm@unime.it (S.P.P.G.); 2 Institute of Translational Pharmacology of the National Research Council, Via Ugo La Malfa, 153, 90146 Palermo, Italy; salvatore.papasergi@cnr.it; 3 Department of Occupational and Environmental Medicine, Epidemiology and Hygiene, Italian Workers’ Compensation Authority (INAIL), Contrada Ficarella, 88046 Lamezia Terme, Italy; 4 Complex Operational Unit of Clinical Pathology, Papardo Hospital, 98158 Messina, Italy; danielecaruso@aopapardo.it

**Keywords:** *Pseudomonas aeruginosa*, osmotic and nutritional stress, biofilm formation, quorum sensing (las/rhl systems), phenazine regulation

## Abstract

*Pseudomonas aeruginosa* is an opportunistic pathogen capable of forming antibiotic-resistant biofilms, contributing to persistent infections and treatment failure. Environmental factors such as osmolarity and nutrient availability are known to influence biofilm formation and virulence. In this study, we investigated the effects of NaCl depletion and glutamine supplementation on biofilm production in three *P. aeruginosa* strains: the laboratory strain ATCC 27853 and two clinical isolates with distinct antibiotic resistance profiles and phenazine production patterns (*P. aeruginosa* Pr, pyorubrin-producing, and *P. aeruginosa* Pc, pyocyanin-producing). Bacteria were cultured in standard Luria–Bertani (LB) medium, LB without NaCl, and LB in which yeast extract was replaced by glutamine. For each strain and condition, we assessed growth kinetics, phenazine production, and biofilm formation. Biofilm development was quantified via XTT assays and compared to secondary metabolite profiles. NaCl removal did not substantially affect growth, whereas glutamine supplementation reduced growth, especially in the laboratory strain. Both conditions modulated secondary metabolite production and biofilm formation in a strain-specific manner. In *P. aeruginosa* ATCC 27853, NaCl depletion significantly increased pyoverdine, pyocyanin, and QS gene expression, while biofilm formation showed significant differences only at 72 h; in contrast, glutamine supplementation affected only pyoverdine. A similar trend was observed in the clinical strain *P. aeruginosa* Pc, although NaCl depletion did not significantly impact pyoverdine production but already enhanced biofilm formation at 48 h. In *P. aeruginosa* Pr, only glutamine appeared to alter the considered parameters, increasing pyoverdine production while reducing pyocyanin and biofilm levels, although the absence of NaCl also negatively impacted biofilm formation. These findings highlight the impact of osmotic and nutritional signals on *P. aeruginosa* virulence traits.

## 1. Introduction

Chronic bacterial infections are among the most challenging conditions in clinical practice, often associated with significant morbidity and mortality [1,2]. A prominent player in these infections is *Pseudomonas aeruginosa* (*P. aeruginosa*), an opportunistic pathogen capable of establishing persistent infections [3,4] and classified among the ESKAPE pathogens due to its high levels of antibiotic resistance [5]. *P. aeruginosa* is responsible for a substantial proportion of nosocomial infections, including ventilator-associated pneumonia, urinary tract infections, bloodstream infections, and catheter-related infections [6,7,8]. Additionally, it is a leading cause of chronic lung infections in individuals with cystic fibrosis, contributing to progressive pulmonary decline [9,10]. Its pathogenicity is largely enhanced by its ability to produce biofilms—complex, three-dimensional microbial communities encased in a self-produced extracellular matrix [11]. This structure not only provides physical protection from external threats, such as antibiotics and the host immune system, but also creates a microenvironment that promotes bacterial survival and adaptability under harsh conditions. Biofilm formation by *P. aeruginosa* is a well-documented contributor to its virulence and resistance mechanisms [12,13,14,15]. Infections caused by this pathogen, particularly in the respiratory tract, wounds, and medical devices, often become chronic due to its ability to resist antimicrobial treatments and evade immune responses [16]. The resilience of biofilms is partly attributed to the spatial heterogeneity within these structures. Oxygen and nutrient gradients, differential metabolic activity, and stress-induced responses collectively enable *P. aeruginosa* to survive otherwise lethal conditions [17,18,19]. The clinical implications of these biofilm properties are profound, as they necessitate innovative therapeutic approaches that target biofilm-specific processes. One environmental factor that significantly influences biofilm formation and architecture is osmotic stress. In clinical settings, *P. aeruginosa* often encounters varying osmolarity levels, particularly in environments like cystic fibrosis (CF) airways or infected wounds [9]. Osmotic stress arises when the extracellular osmolarity deviates from physiological levels, imposing selective pressure on microbial populations. This stress triggers a range of adaptive responses in *P. aeruginosa*, including alterations in gene expression, metabolic pathways, and biofilm production [20,21,22]. Studies have shown that osmotic stress can modify the structure of biofilms, potentially rendering them more susceptible to antibiotic penetration or, conversely, enhancing their resistance by inducing protective mechanisms [23]. The ability of *P. aeruginosa* to thrive under osmotic stress is closely linked to its nitrogen metabolism, particularly through the utilization of amino acids such as glutamine [24]. Amino acids not only serve as fundamental building blocks for protein synthesis but also act as crucial nitrogen sources and signaling molecules in microbial physiology [25]. Among these, glutamine plays a pivotal role in regulating biofilm formation and virulence in *P. aeruginosa* [24]. When readily available, glutamine can modulate key cellular pathways related to energy production, osmoprotection, and extracellular matrix synthesis—ultimately affecting biofilm architecture and resilience [26]. Together, these findings highlight a complex interplay between nutrient availability, environmental stressors, and microbial pathogenicity in P. aeruginosa. Understanding the role of glutamine and osmotic stress in biofilm formation provides critical insights into the pathophysiology of P. aeruginosa infections. The extracellular matrix of biofilms is primarily composed of polysaccharides, proteins, and extracellular DNA (eDNA), all of which contribute to its structural integrity and protective functions [22]. The availability of nitrogen sources such as glutamine can influence the synthesis of these components, altering biofilm architecture and potentially affecting susceptibility to antibiotics [27]. Moreover, osmotic stress may enhance the expression of stress-response genes, further reinforcing biofilm resilience. From a clinical perspective, these interactions have significant implications for the treatment of *P. aeruginosa* infections. Chronic infections, particularly in immunocompromised patients, are notoriously difficult to eradicate due to the dual challenges of antibiotic resistance and biofilm-mediated protection [28]. Standard antibiotic therapies are often ineffective against biofilms, as the extracellular matrix impedes drug diffusion and creates a sanctuary for dormant bacterial cells [29]. Addressing the influence of environmental factors like osmotic stress and nutrient availability on biofilm dynamics may open new avenues for therapeutic interventions. One promising area of research is the development of adjuvant therapies that exploit biofilm vulnerabilities induced by osmotic stress. For example, hypertonic saline solutions have been investigated for their potential to disrupt biofilm structure and enhance antibiotic efficacy [30,31]. Similarly, targeting nitrogen metabolism pathways, including those involving glutamine, may provide a novel strategy for weakening biofilm defenses [32,33]. These approaches underscore the importance of integrating a deeper understanding of microbial physiology into clinical practice. The role of *P. aeruginosa* biofilms in chronic infections extends beyond their intrinsic resistance mechanisms. Biofilm-associated infections also exacerbate host tissue damage and inflammation, compounding the clinical burden [19]. In the context of CF, for example, *P. aeruginosa* biofilms in the airways contribute to persistent inflammation, impaired mucociliary clearance, and progressive lung damage [10]. These effects are partly mediated by the pathogen’s ability to modulate the host immune response, often dampening antibacterial defenses through mechanisms linked to osmotic stress and amino acid metabolism. Therefore, biofilm formation by *P. aeruginosa* represents a formidable challenge in the management of chronic infections. In this work, the influence of osmotic stress and glutamine availability on biofilm production and secondary metabolite synthesis was evaluated, with the aim of understanding microbial adaptability and identifying potential targets for therapeutic intervention.

## 2. Materials and Methods

### 2.1. Bacteria Strain, Media, and Growth Conditions

*P. aeruginosa ATCC 27853* was purchased from the American Type Culture Collection (LGC Promochem, Milan, Italy). In addition to this reference strain, two clinical isolates—labelled *P. aeruginosa* Pr (*pyorubrin producer*) and *P. aeruginosa* Pc (*pyocyanin producer*)—were included based on their phenotypic characteristics.

The identification of clinical isolates of *P. aeruginosa* from bronchoalveolar lavage was carried out by means of mass spectrometric analysis MALDI-ToF (Matrix-Assisted Laser Desorption/Ionization Time of Flight), according to the experimental instructions provided by the manufacturer (Bruker Daltonics, Milano, Italy). For final identification, standard Bruker interpretative criteria were applied with a score ≥ 2.0 for species assignment, and scores between 1.7 and 2.0 for genus-level identification. Scores below 1.7 were considered unreliable.

All experimental conditions were based on Luria–Bertani (LB) broth, which served as both the basal medium and the standard control. LB is a nutrient-rich formulation widely used to support robust bacterial growth under laboratory conditions. To investigate the role of sodium chloride, a modified version of the medium lacking NaCl (LB–NaCl) was prepared, containing only tryptone and yeast extract. This formulation was used to assess the impact of osmotic strength on bacterial growth and physiological responses. Otherwise, to evaluate the effect of a defined nitrogen source, yeast extract was replaced with L-glutamine (LB–YE/GLn). Specifically, the medium contained per liter: 10 g of tryptone, 10 g of NaCl, and 50 mL of sterile-filtered L-glutamine solution (200 mM), yielding a final glutamine concentration of 10 mM. While this formulation includes L-glutamine as a chemically defined nitrogen source, it must be noted that the presence of tryptone—a complex hydrolysate—prevents the medium from being fully defined. Because glutamine is heat-sensitive, it was added to the autoclaved and cooled sterile medium to reach a final concentration of 10 mM.

All culture media were adjusted to pH 7.0 and sterilized by autoclaving at 121 °C for 20 min to ensure complete elimination of microbial contaminants.

### 2.2. Antimicrobial Resistance Profiles of P. aeruginosa Strains

The antimicrobial resistance profiles of the clinical *P. aeruginosa* isolates, compared to a laboratory reference strain, were assessed using the VITEK 2 Compact system (bioMérieux, Paris, France), following the manufacturer’s instructions. This automated system monitors the growth and metabolic activity of microorganisms in wells contained within specialized test cards. Each card features 64 wells, with individual substrates designed to assess various metabolic activities, including acidification, alkalinization, enzymatic hydrolysis, and growth in the presence of inhibitory substances. The aim of this analysis was to evaluate and compare the susceptibility patterns of the strains to better understand differences in their resistance profiles.

### 2.3. Bacterial Growth Assessment

The growth of the three *P. aeruginosa* strains—ATCC 27853 (reference strain), and the two clinical isolates *P. aeruginosa* Pr (pyorubrin producer) and Pc (pyocyanin producer)—was spectrophotometrically evaluated. Specifically, overnight cultures of the three strains were first adjusted to a turbidity equivalent to 0.5 McFarland, then inoculated into sterile tubes containing one of the three previously described culture media: LB (control), LB without sodium chloride (LB-NaCl), and the glutamine-substituted medium (LB-YE/GLn). Each experimental condition was prepared in sterile 50 mL Falcon tubes with a final volume of 10 mL and inoculated with the corresponding *P. aeruginosa* strain at a concentration of 10^7^ CFU/mL. Cultures were incubated at 37 °C with shaking at 150 rpm for 24 h. Growth was monitored by colony-forming unit (CFU) assays, performed hourly during the first 8 h to generate time-course growth curves, and subsequently at 24, 48, and 72 h. At each time point and for each condition, serial dilutions of 100 μL aliquots were plated in triplicate on appropriate agar medium, and colonies were counted after incubation to determine viable cell numbers.

### 2.4. Pyoverdine Assay

Pyoverdine production from *P. aeruginosa* was quantified according to the protocol reported by Fekete-Kertész et al. [34]. Specifically, 800 μL from each experimental condition was transferred into sterile microtubes and centrifuged at 6000× *g* for 10 min. The resulting supernatant was then transferred to a 96-well plate and analyzed using a Perkin Elmer Wallac 1420 Victor2 Microplate Reader (Turku, Finland). Background fluorescence from fresh, uninoculated media was subtracted. Pyoverdine production was measured by fluorescence with excitation/emission wavelengths set at 485 nm/535 nm.

### 2.5. Phenazine Assay

Phenazine, pyocyanin, and pyorubrin production from *P. aeruginosa* was quantified according to the protocol reported by Zanni et al. [35]. Specifically, 800 μL from each experimental condition was transferred into sterile microtubes and centrifuged at 6000× *g* for 10 min. Centrifugation was necessary to remove bacterial cells and avoid their interference with the spectrophotometric absorbance readings. The resulting supernatant was transferred into a 96-well plate at 200 μL per well, in triplicate for each condition, and analyzed using a Perkin Elmer Wallac 1420 Victor2 Microplate Reader (Turku, Finland). Background absorbance from fresh, uninoculated media was subtracted. Pyocyanin production was quantified by measuring absorbance at 405 nm. Measurements were performed at 48 and 72 h, corresponding to the time points at which maximum pigment production was observed. These time points were also selected because they coincide with key stages in *P. aeruginosa* biofilm development—48 h representing an advanced maturation phase and 72 h corresponding to the establishment of mature biofilms—allowing us to correlate pigment synthesis with biofilm formation capacity under the tested conditions.

### 2.6. Biofilm Assay

The ability to produce biofilm was evaluated in 96-well polystyrene microtiter plates according to the procedure described by Coffey and Anderson [36] with some modifications. Overnight cultures of *P. aeruginosa* ATCC 27853 and the clinical isolates *P. aeruginosa* Pr and Pc were first adjusted to a turbidity equivalent to 0.5 McFarland in the three different culture media previously described. Bacterial suspensions were then dispensed into flat-bottom 96-well polystyrene microtiter plates at 200 μL per well, in triplicate for each experimental condition. The plates were incubated under static conditions at 37 °C for 48 and 72 h to allow for the development of mature biofilms. Following incubation, non-adherent (planktonic) bacteria were removed by washing each well three times with 300 μL of phosphate-buffered saline (PBS, pH 7.4). Each washing step consisted of filling the well, gently shaking for 5 min, and then removing the liquid. After the final wash, 200 μL of PBS and 50 μL of XTT solution (prepared according to the manufacturer’s instructions) were added to each well. The plates were incubated in the dark at 37 °C for 3 h. Subsequently, gentle agitation was applied, and absorbance was measured at 490 nm using a Perkin Elmer Wallac 1420 Victor2 Microplate Reader (Turku, Finland). Absorbance values were corrected by subtracting the background levels of the corresponding uninoculated media.

Biofilm reduction (Br) under each cultural condition was calculated as a percentage relative to the LB control using the following equation:

$$Br=(\frac{A-B}{A})\times 100$$

where *A* and *B* are the OD_490_ from CTR (LB) and varied cultural conditions (LB-NaCl or LB-YE/GLn).

### 2.7. RNA Isolation

Total RNA was extracted from bacterial cultures using TRIzol^®^ Reagent (Thermo Fisher Scientific, Milano, Italy), with protocol modifications to optimize RNA recovery from dense cell suspensions. Bacteria were transferred to RNase-free microcentrifuge tubes during the early stationary phase (12–16 h), corresponding to the peak expression of *lasI* and *rhl* genes, and associated with maximal production of quorum-sensing (QS) signals and initiation of biofilm formation. Cells were collected by centrifugation at 8000× *g* for 8 min at 4 °C. The pellet was resuspended in 1 mL of TRIzol and disrupted by vortex for 5 min and sonication for 2 min. Samples were incubated at room temperature for 15 min. Chloroform (200 µL) was added, mixed vigorously, and incubated for 3 min before centrifugation at 12,000× *g* for 15 min at 4 °C. The aqueous phase was transferred to a new tube, mixed with an equal volume of 100% ethanol, and centrifuged at 12,000× g for 10 min. The RNA pellet was washed with 75% ethanol, centrifuged, air-dried, and resuspended in RNase-free water. RNA concentration and purity were assessed by spectrophotometry.

### 2.8. Reverse Transcription PCR (RT-PCR)

Complementary DNA (cDNA) was synthesized from total RNA using the ImProm-II™ Reverse Transcription System (Promega, Milano, Italy), following the manufacturer’s protocol [37]. Briefly, 1 µg of total RNA was used as input for each 20 µL reverse transcription reaction. RNA was first incubated with 0.5 µg of Random Hexamers primers in a total volume of 5 µL. The mixture was heated to 70 °C for 5 min and then immediately placed on ice for at least 5 min to allow primer annealing. Subsequently, 15 µL of the reverse transcription master mix was added to each reaction tube, containing Buffer, MgCl_2_ to a dNTP mix (10 mM each), and 1 µL of reverse transcriptase. RNasin^®^ Ribonuclease Inhibitor (1 µL, 40 U/µL) was also included to prevent RNA degradation. Reverse transcription was carried out in a thermal cycler with the following conditions: 25 °C for 5 min (primer extension initiation), 42 °C for 60 min, and 70 °C for 15 min. The resulting cDNA was stored at −20 °C and subsequently used as a template for quantitative PCR analysis.

### 2.9. Primer Design and Quantitative Real-Time PCR

mRNA sequences corresponding to key quorum-sensing (QS) genes *lasI*, *lasR*, *rhlI*, and *rhlR* were retrieved from the NCBI Reference Sequence (RefSeq) database, and specific primers were designed using the Primer-BLAST tool. The final primer sequences were synthesized by a commercial supplier and used in real-time PCR (qRT-PCR) to validate the differential expression levels in samples.

Gene expression was quantified using SsoAdvanced™ Universal SYBR^®^ Green Supermix (Bio-Rad, Milano, Italy) following the manufacturer’s protocol [38]. Each 10 μL reaction contained 5 μL SYBR Green Supermix, 0.4 μL each of forward and reverse primers, 2 μL of cDNA, and nuclease-free water. Reactions were run in triplicate under the following conditions: 95 °C for 2 min, followed by 40 cycles of 95 °C for 3 s and 60 °C for 30 s.

No-template controls (NTCs) were included for each primer set. Primer efficiency was verified (90–110%), and gene expression was calculated using the 2^−ΔΔCt method. The enzyme Δ1-pyrroline 5-carboxylate reductase involved in proline biosynthesis (*proC*) was used as the endogenous reference gene.

### 2.10. Statistical Analysis

All results are expressed as the arithmetic mean ± standard deviation (SD) from three independent biological replicates and are reported as a percentage relative to the control condition (LB). Statistical analysis of differences between experimental groups was performed using Tukey’s multiple comparisons test.

## 3. Results

In this study, the laboratory strain *P. aeruginosa* ATCC 27853 and two clinical isolates were used. These clinical strains were selected based on their distinctive pigment production and antibiotic resistance profiles, which differed from those of the ATCC 27853 strain. Specifically, when cultured on Cetrimide agar, P. aeruginosa Pr exhibited an intense red pigmentation associated with pyorubrin production, whereas P. aeruginosa Pc displayed the characteristic blue-green pigmentation indicative of pyocyanin production. Table 1 shows the Minimum Inhibitory Concentrations (MICs, in mg/L) of six common antimicrobial agents used against *P. aeruginosa*. The interpretations of MIC values were based on the EUCAST Clinical Breakpoint guidelines (https://www.eucast.org/clinical_breakpoints, accessed on 1 July 2025).

Results indicated that reference strain *P. aeruginosa* ATCC 27853 showed consistent susceptibility compared to clinical isolates *P. aeruginosa* Pc and *P. aeruginosa* Pr. In particular, *P. aeruginosa Pr* exhibited higher resistance to Imipenem and Meropenem, while *P. aeruginosa Pc* towards Cefepime, Ceftazidime, and Piperacillin/Tazobactam. On the other hand, all strains were susceptible to amikacin. VITEK analysis revealed that *P. aeruginosa* Pr exhibited acquired penicillinase and carbapenem resistance (impermeability), whereas *P. aeruginosa* Pc displayed high-level cephalosporinase activity.

**Figure 1:**
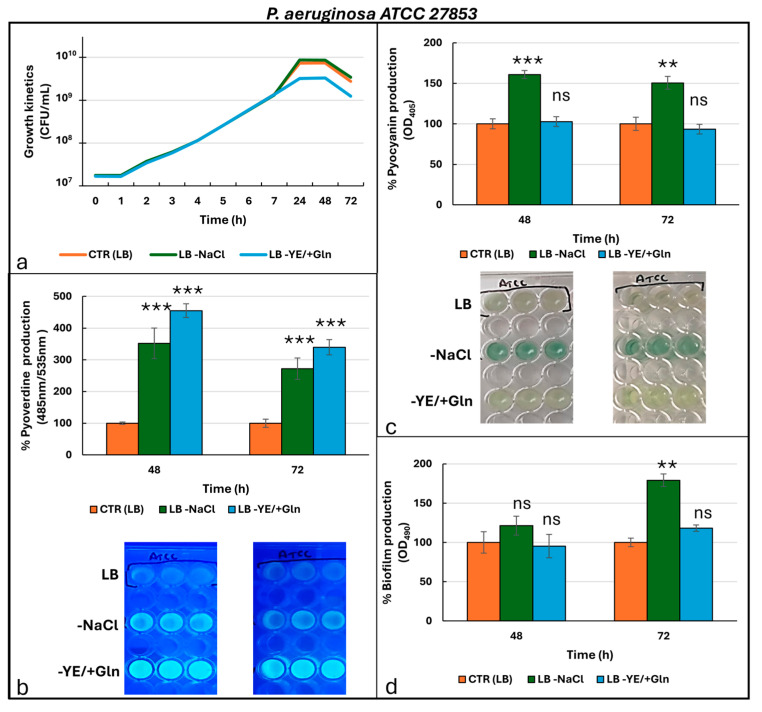
Growth kinetics (**a**) and production of pyoverdine (**b**), pyocyanin (**c**), and biofilm (**d**) by *P. aeruginosa* ATCC 27853 under different culture conditions. Photos in the frames in (**b**,**c**) are representative of the production of pyoverdine (under UV-light at 366 nm) and pyocyanin, respectively. For the ANOVA test, Tukey’s post hoc test for multiple comparisons was used (Tables S1–S4), identifying with one (*), two (**), and three (***) asterisks, adjusted *p*-value < 0.05, 0.01, and 0.001, respectively. ns: not statistically significant.

Figure 1 shows the result for the laboratory strain *P. aeruginosa* ATCC 27853.

**Figure 2:**
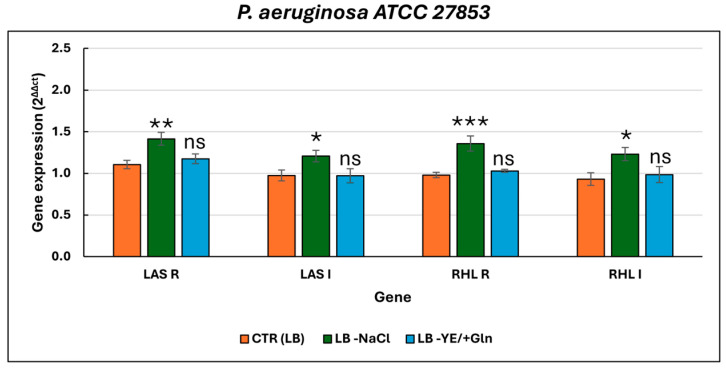
Relative expression levels of key quorum-sensing genes (*lasI*, *lasR*, *rhlI*, and *rhlR*) in *P. aeruginosa* ATCC 27853 under different culture conditions. For the ANOVA test, Tukey’s post hoc test for multiple comparison was used (Table S5), identifying with one (*), two (**), and three (***) asterisks, adjusted *p*-value < 0.05, 0.01, and 0.001, respectively. ns: not statistically significant.

The growth kinetics (Figure 1a) showed a steady increase in cell growth during the first 7 h, with no significant differences among the culture conditions. At 24 h, the strains reached their maximum growth density, although the LB -YE/+Gln condition produced a lower maximum compared to LB and LB -NaCl. Between 24 and 48 h, cell density remained stable, followed by a slight decline at 72 h. During the 24–72 h interval, growth in LB -YE/+Gln was significantly lower than in LB and LB -NaCl at all time points (*p* < 0.0001), highlighting the restrictive nature of this medium (Table S1). Differences between LB and LB -NaCl were generally less pronounced and often not statistically significant. The production of secondary metabolites and biofilms was specifically analyzed at two predefined time intervals, namely after 48 and 72 h of incubation. Pyoverdine production (Figure 1b) was highest in LB -YE/+Gln at 48 h, with significantly greater levels compared to LB -NaCl and the control (Table S2). Although pyoverdine levels decreased across all conditions after 72 h, LB -YE/+Gln continued to exhibit the highest production among the tested media. Pyocyanin production (Figure 1c) peaked in LB -NaCl at 48 h, with significantly lower levels observed in both the LB and LB -YE/+Gln conditions (Table S3). While pyocyanin production in LB -NaCl declined slightly at 72 h, it still retained the highest output. Conversely, LB -YE/+Gln exhibited consistent but notably lower production over time. Biofilm formation (Figure 1d) was most robust in LB -NaCl at 72 h, with values only slightly lower at 48 h. Both the control LB and LB -YE/+Gln produced significantly lower biofilm levels compared to LB -NaCl condition, with the difference becoming more pronounced at 72 h (Table S4). Quantitative gene expression analysis revealed a significant upregulation of the quorum-sensing (QS) genes *lasI*, *lasR*, *rhlI*, and *rhlR* compared to expression levels in standard LB medium (Figure 2).

Specifically, when the bacterial strain was grown in the absence of NaCl (LB–NaCl), *rhlR* showed the highest induction, followed by *lasR* and *lasI*, along with *rhlI*. In contrast, when L-glutamine was added in place of yeast extract (LB–YE/+Gln), the expression levels of these genes remained comparable to the standard control condition (LB) and were significantly lower than those observed in the NaCl-depleted medium (Table S5).

**Figure 3:**
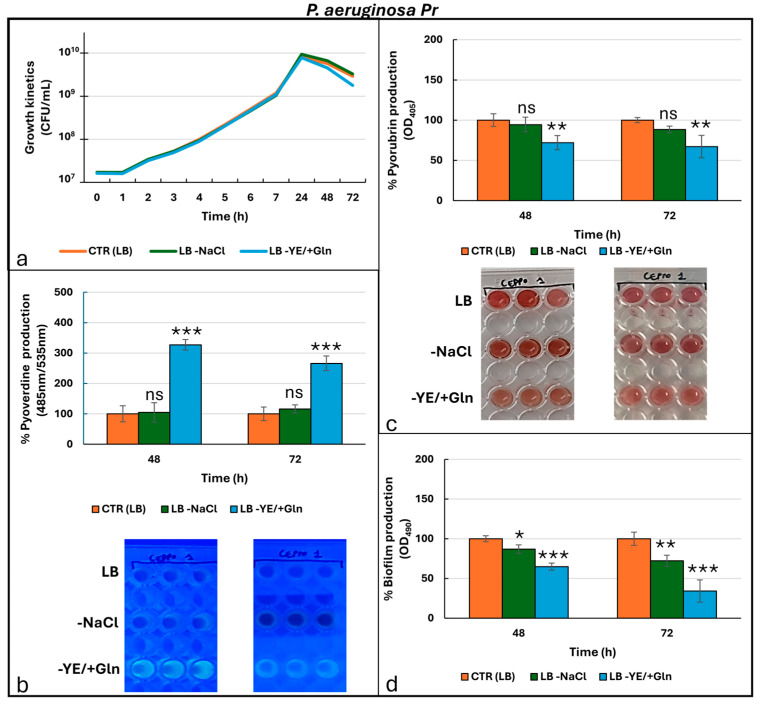
Growth kinetics (**a**) and production of pyoverdine (**b**), pyorubrin (**c**), and biofilm (**d**) by *P. aeruginosa Pr* under different culture conditions. Photos in the frames in (**b**,**c**) are representative of the production of pyoverdine (under UV-light at 366 nm) and pyorubrin, respectively. For the ANOVA test, Tukey’s post hoc test for multiple comparisons was used (Tables S6–S9), identifying with one (*), two (**), and three (***) asterisks, adjusted *p*-value < 0.05, 0.01, and 0.001, respectively. ns: not statistically significant.

Figure 3 shows results for clinical strain *P. aeruginosa Pr* (pyorubrin producer).

**Figure 4:**
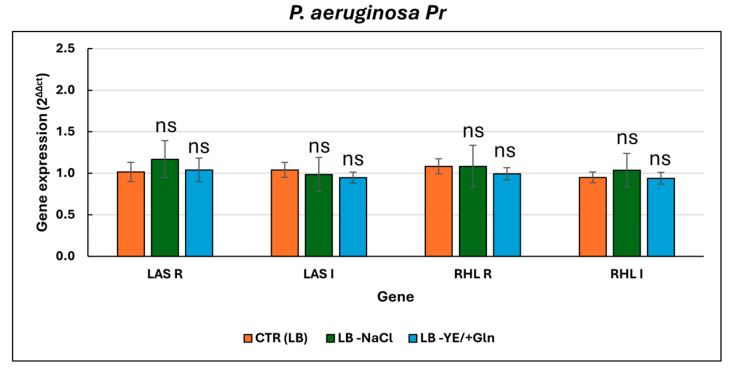
Relative expression levels of key quorum-sensing genes (*lasI*, *lasR*, *rhlI*, and *rhlR*) in *P. aeruginosa* Pr under different culture conditions. For the ANOVA test, Tukey’s post hoc test for multiple comparisons was used (Table S10), identifying with one (*), two (**), and three (***) asterisks, adjusted *p*-value < 0.05, 0.01, and 0.001, respectively. ns: not statistically significant.

The growth kinetics (Figure 3a) of *P. aeruginosa Pr* showed a trend similar to that observed for the laboratory strain; no significant difference was detected (Table S6). Pyoverdine production was strongly influenced by LB -YE/+Gln, with peak values observed at 48 h (Figure 3b, Table S7). This trend continued through 72 h, showing only a slight decline. Comparatively, the LB -NaCl condition resulted in slightly higher values than the LB condition across the time intervals analyzed. However, these differences were not statistically significant (Table S7), suggesting that the removal of NaCl may have had a minimal but non-critical impact on the observed outcomes. Regarding the production of phenazine-related products, the clinical strain consistently exhibited high levels of pyorubrin production across all cultural conditions tested (Figure 3c). However, under the LB -YE/+Gln condition, production levels significantly deviated from those observed in the LB condition, suggesting a potential role for Gln in inhibiting this metabolite’s production (Table S8). Additionally, the absence of NaCl resulted in lower pyoverdine production levels compared to the control condition, although these differences were not statistically significant (Table S8). Finally, regarding biofilm production (Figure 3d), the data indicated a direct correlation with pyorubrin production. In fact, under the LB -YE/+Gln condition, biofilm production was significantly reduced at both 48 and 72 h (Table S9). Furthermore, the LB -NaCl condition also negatively impacted biofilm production, although the differences compared to the control were only significant at 72 h (Table S9), suggesting a time-dependent effect of NaCl removal on biofilm formation. In contrast to the laboratory strain (*P. aeruginosa* ATCC 27853), *P. aeruginosa* Pr did not show differential expression of *lasI*, *lasR*, *rhlI*, or *rhlR* across the three tested growth conditions (Figure 4).

Gene expression levels in this strain remained stable and comparable across all media, with no statistically significant fold changes observed (Table S10). This suggests that the QS regulatory circuit in *P. aeruginosa* Pr is either less responsive to environmental ionic or metabolic changes, or is constitutively active under biofilm-forming conditions, regardless of external medium composition.

**Figure 5:**
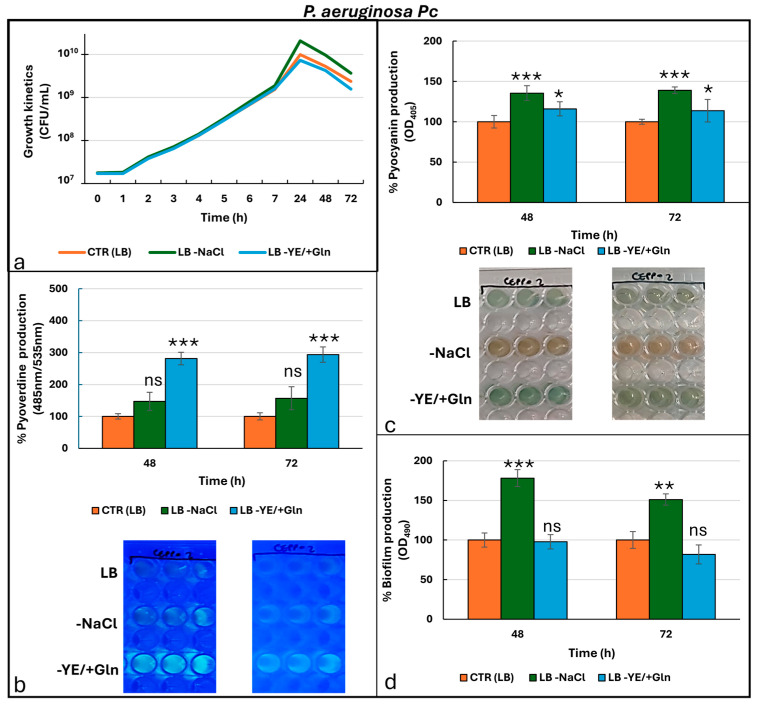
Growth kinetics (**a**) and production of pyoverdine (**b**), pyocyanin (**c**), and biofilm (**d**) by *P. aeruginosa Pc* under different culture conditions. Photos in the frames in (**b**,**c**) are representative of the production of pyoverdine (under UV-light at 366 nm) and pyocyanin, respectively. For the ANOVA test, Tukey’s post hoc test for multiple comparisons was used (Tables S11–S14), identifying with one (*), two (**), and three (***) asterisks, adjusted *p*-value < 0.05, 0.01, and 0.001, respectively. ns: not statistically significant.

Figure 5 shows results for clinical strain *P. aeruginosa Pc* (pyocyanin producer).

**Figure 6:**
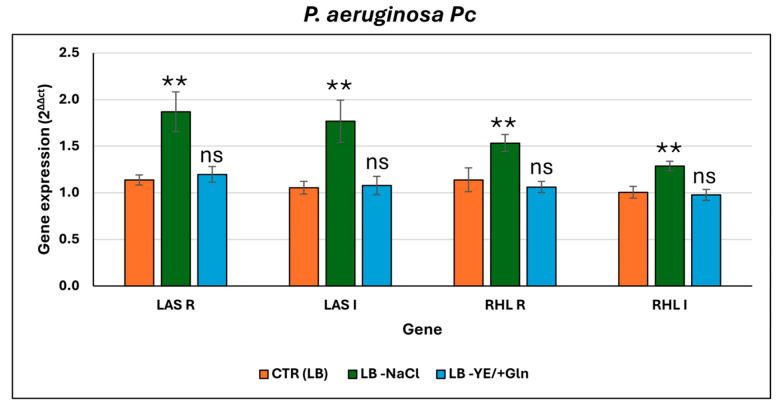
Relative expression levels of key quorum-sensing genes (*lasI*, *lasR*, *rhlI*, and *rhlR*) in *P. aeruginosa* Pc under different culture conditions. For the ANOVA test, Tukey’s post hoc test for multiple comparisons was used (Table S15), identifying with one (*), two (**), and three (***) asterisks, adjusted *p*-value < 0.05, 0.01, and 0.001, respectively. ns: not statistically significant.

*P. aeruginosa Pc* exhibited the highest overall growth compared to the other strains tested, with particularly notable results observed under condition (Figure 5a). Unlike the other strains analyzed in the present study, growth under the LB -NaCl condition was significantly increased in comparison to the other tested conditions (Table S11). In contrast, no significant difference in growth was detected for the LB and LB -YE/+Gln condition, indicating that glutamine does not substantially affect the strain’s growth under the given conditions (Table S11). The production of secondary metabolites and biofilms also followed a trend that was quite similar to that observed in the laboratory strain. Regarding pyoverdine (Figure 5b), *P. aeruginosa* Pc strain showed a significant increase in production across all time points analyzed when cultured in the LB -YE/+Gln condition (Table S12). Although the LB -NaCl condition also appeared to induce an increase in pyoverdine production, no statistically significant differences were detected compared to the standard LB condition, suggesting that the removal of NaCl had a limited effect under these specific circumstances (Table S12). Pyocyanin synthesis (Figure 5c) was significantly pronounced in the LB -NaCl condition, although relatively substantial production levels were also observed in both the LB and LB -YE/+Gln conditions (Table S13). Visually, cultures in the LB -NaCl condition underwent a notable colorimetric shift, changing from green to dark orange, ascribable to an increase in reduced pyocyanin. This accumulation could be attributed to a combination of accelerated growth and a resulting depletion of bioavailable oxygen in the culture medium compared to the other ones. Also for *P. aeruginosa Pc*, biofilm production was directly proportional to the production of phenazines (Figure 5d). Specifically, biofilm formation reached its peak in the LB -NaCl condition at 48 h and remained significantly elevated at 72 h (Table S14). In contrast, the LB and LB -YE/+Gln conditions were associated with much lower biofilm production capacities. These findings confirm that salt depletion serves as a strong stimulus for biofilm formation in *P. aeruginosa* Pc, likely by altering environmental and physiological factors conducive to biofilm development, such as osmotic stress or nutrient availability. A similar expression pattern to the laboratory strain was observed in *P. aeruginosa* Pc (Figure 6).

Specifically, *lasI*, *lasR*, *rhlI*, and *rhlR* were also upregulated in the LB -NaCl condition, compared to both LB and LB -YE/+Gln conditions. However, the magnitude of gene induction in the clinical strain was consistently higher than in the reference laboratory strain under the LB -NaCl (Table S15). Fold changes in *P. aeruginosa* Pc strain ranged from approximately 0.5- to 1-fold, depending on the gene, suggesting a strain-specific increase in sensitivity or responsiveness to ionic stress in the context of QS activation.

To clarify whether the responses to different environmental conditions represent conserved species-level traits or, alternatively, strain-specific adaptations, a comparative analysis among the three *P. aeruginosa* strains was also introduced (Table 2).

The comparative analysis of *P. aeruginosa* ATCC27853, *P. aeruginosa* Pr, and *P. aeruginosa* Pc under different culture conditions showed that all strains had similar cell viability with maximum CFU/mL at 24 h and a decline at later time points. *P. aeruginosa* Pc consistently reached the highest cell densities, while *P. aeruginosa* ATCC27853 and *P. aeruginosa* Pr displayed slightly lower but comparable levels. The removal of NaCl or the addition of glutamine did not substantially alter growth, indicating stable viability across strains. In terms of pyoverdine production, *P. aeruginosa* ATCC27853 was the most efficient producer, particularly in glutamine-supplemented medium, while *P. aeruginosa* Pc showed intermediate values, and *P. aeruginosa* Pr remained the lowest. An opposite trend was observed for pyocyanin production: in fact, *P. aeruginosa* Pr and *P. aeruginosa* Pc produced higher levels than *P. aeruginosa* ATCC27853 in LB, and their production increased further in NaCl-depleted medium, whereas glutamine supplementation reduced synthesis in all strains, most markedly in *P. aeruginosa* Pr. Biofilm formation also varied: at 48 h *P. aeruginosa* ATCC27853 produced the strongest biofilm, but at 72 h *P. aeruginosa* Pc surpassed it in NaCl-depleted conditions, while *P. aeruginosa* Pr remained lower. Glutamine supplementation reduced biofilm formation in both clinical isolates, whereas *P. aeruginosa* ATCC27853 maintained moderate levels.

## 4. Discussion

This study investigated the metabolic pathways related to virulence and environmental adaptability strategies of three distinct *P. aeruginosa* strains under different culture conditions. Specifically, we used the laboratory strain *P. aeruginosa* ATCC 27853 as a standard reference, along with two clinical isolates selected for their differing antibiotic resistance profiles and distinct phenazine production. The antibiotic resistance profiles of the tested isolates were included to characterize the clinical strains, highlight their distinct origins, and provide valuable context for interpreting their metabolic behaviors. Results from Table 1 indicated that the laboratory strain displayed consistent susceptibility, particularly to Meropenem and Amikacin (classified as S), with intermediate susceptibility (I) to other tested antibiotics like Imipenem, Cefepime, and Ceftazidime. Conversely, the clinical isolates demonstrated higher levels of resistance, emphasizing their pathogenic adaptability. In fact, *P. aeruginosa* Pr exhibited resistance to both Imipenem (MIC > 8 mg/L) and Meropenem (MIC > 8 mg/L), indicating its reduced susceptibility to carbapenems, a class of antibiotics frequently reserved for treating severe or multidrug-resistant bacterial infections [39]. These antibiotics are considered “last-resort treatments” because they are often employed only when other antibiotic options have failed, particularly in life-threatening situations such as ventilator-associated pneumonia or bloodstream infections caused by highly resistant bacteria [40]. Otherwise, *P. aeruginosa* Pc demonstrated significant resistance to third- and fourth-generation cephalosporins, such as Cefepime (MIC 16 mg/L) and Ceftazidime (MIC 32 mg/L), as well as to Piperacillin/Tazobactam (MIC > 64 mg/L) [41,42]. These antibiotics are among the most commonly used antipseudomonal agents for treating severe infections, such as pneumonia, bloodstream infections, and urinary tract infections [43]. Further insights from VITEK analysis provided a mechanistic explanation for these resistance profiles. *P. aeruginosa Pr* exhibited acquired penicillinase activity along with carbapenem resistance due to reduced membrane permeability (impermeability), which is consistent with its elevated MICs for Imipenem and Meropenem. In contrast, *P. aeruginosa Pc* displayed high-level cephalosporinase activity, explaining its marked resistance to third- and fourth-generation cephalosporins. These enzymatic patterns highlight distinct adaptive strategies between the clinical isolates, reflecting how *P. aeruginosa* can employ different resistance mechanisms—either through enzymatic degradation or membrane modifications—to survive in the antibiotic-rich clinical environment [44,45]. The heterogeneous antibiotic resistance profiles of the tested isolates provide valuable context for interpreting their metabolic behaviors. In particular, strains with distinct resistance patterns may adopt different stress adaptation strategies, which could influence their ability to respond to environmental cues such as elevated NaCl or the presence of a specific nutrient source. These metabolic and stress-response adaptations may, in turn, affect biofilm production, suggesting that antibiotic resistance and biofilm-forming capacity are interconnected traits that help *P. aeruginosa* survive under challenging conditions. The three nutritional conditions used—LB medium (CTR), low-salt medium (LB -NaCl), and yeast extract-free medium supplemented with glutamine (LB -YE/+Gln)—highlighted key strategies in the metabolic adaptability and environmental responsiveness of *P. aeruginosa* strains with varied origins and phenotypic characteristics. Each condition emphasized distinct aspects of physiological behavior (growth kinetics) and secondary metabolism (pyoverdine, phenazines, and biofilm production), providing valuable insights into the virulence mechanisms and environmental adaptability of *P. aeruginosa*. About secondary metabolism, all experimental conditions were monitored at two time points, 48 and 72 h, to assess the accumulation of pyoverdine and pyocyanin in relation to biofilm development. These time points were specifically selected to capture both the early and more advanced phases of *P. aeruginosa* biofilm formation. Pyoverdine, a siderophore essential for iron acquisition, plays a critical role not only in the early stages of biofilm establishment but also in regulating its maturation. Its progressive increase is commonly associated with enhanced biofilm stability and virulence, making the 72 h mark particularly relevant for evaluating mature biofilm-associated phenotypes [46]. In parallel, pyocyanin production—closely linked to quorum-sensing (QS) and typically initiating as early as 18 h—undergoes a marked increase during the transition to the stationary phase. This redox-active pigment contributes to oxidative stress, host tissue damage, and plays a key role in redox homeostasis and the structural organization of the biofilm matrix [47]. In our experimental setting, we observed that pyoverdine production generally peaked at 48 h and declined thereafter, whereas biofilm formation reached its maximum levels at 72 h, particularly under NaCl depletion. This temporal divergence indicates that the siderophore is mainly involved in the early and intermediate stages of biofilm establishment, while the subsequent consolidation of the biofilm architecture occurs later and is strongly influenced by ionic stress. For this reason, the 72 h time point was selected as the most appropriate to capture both the late-phase metabolite dynamics and the mature biofilm phenotype. The results clearly demonstrate that culture conditions significantly influence the physiology of *P. aeruginosa* ATCC 27853, a widely used laboratory model strain, affecting growth, secondary metabolite production, and biofilm formation. The reduced growth observed in LB -YE/+Gln suggests that glutamine supplementation cannot fully replace the complex nutrients provided by yeast extract, likely limiting essential cofactors needed for optimal proliferation. Interestingly, this condition triggered the highest pyoverdine production, indicating a possible iron-limited state or metabolic shift towards stress responses. Conversely, the LB -NaCl medium, despite being less favorable for growth, promoted the highest pyocyanin levels and biofilm formation, implying that osmotic stress or ionic imbalance may activate QS and redox-sensitive pathways that enhance competitive and persistence traits. These findings may be attributed to the bacterium’s regulatory mechanisms governing nutrient utilization. Recent studies have highlighted the role of the AauR–AauS two-component system in regulating the uptake and metabolism of acidic amino acids, such as glutamate and glutamine [24]. Deletion of the aauR gene impairs the bacterium’s ability to utilize these amino acids effectively, leading to growth limitations under specific nutrient conditions. Additionally, the CbrAB two-component system has been implicated in modulating substrate prioritization through the catabolite repression control pathway, influencing the bacterium’s adaptability to various nutrient environments [48]. These regulatory pathways underscore the complexity of *P. aeruginosa*’s metabolic network and its dependence on specific nutrient conditions for optimal growth. When *P. aeruginosa* ATCC 27853 grows in LB-NaCl, there is a marked upregulation of key QS genes—*lasI*, *lasR*, *rhlI*, and *rhlR*. This suggests that the bacterium perceives salt depletion as a stress cue, likely associated with altered osmolarity or disruption of ionic homeostasis, which in turn activates QS pathways to promote coordinated behaviour and stress adaptation. Notably, when yeast extract was replaced with L glutamine—as a defined nitrogen and energy source—the expression of these genes remained comparable to that observed in standard LB. This indicates that L-glutamine supplementation, despite influencing growth and pyoverdine production, does not significantly impact QS regulation. The consistent upregulation of QS genes in the LB-NaCl, as opposed to the glutamine-supplemented condition, underscores the predominant role of ionic availability over nutrient complexity in modulating QS responses. These findings reinforce the hypothesis that osmotic and ionic signals act as key environmental triggers for the activation of regulatory circuits governing biofilm development and secondary metabolite production. In this context, NaCl depletion may mimic environmental stress conditions, thereby inducing competitive and persistence-related traits through enhanced QS activity.

Comparing the laboratory, *P. aeruginosa* ATCC 27853, with the clinical isolate *P. aeruginosa* Pr, reveals both shared and distinct adaptive responses. Growth kinetics were similar between the two strains across conditions, indicating that the fundamental growth capacity is conserved. Both strains increased pyoverdine production in LB -YE/+Gln, highlighting a common iron-scavenging mechanism triggered under nutrient limitation and L-glutamine supplementation. However, phenazine production differed notably: *P. aeruginosa* ATCC 27853 peaked in pyocyanin production under LB -NaCl, whereas *P. aeruginosa* Pr consistently produced high pyorubrin levels across conditions, with a marked suppression in LB -YE/+Gln. This suggests that glutamine may inhibit pyorubrin synthesis through mechanisms such as nitrogen catabolite repression or altered redox balance, which may be regulated differently in clinical isolates. Regarding biofilm formation, *P. aeruginosa* ATCC 27853 formed the most robust biofilms in LB -NaCl alongside high pyocyanin levels, while *P. aeruginosa* Pr showed reduced biofilm formation in both LB -YE/+Gln and LB -NaCl, closely correlating with decreased pyorubrin production. This indicates that in clinical strains, phenazine-mediated redox signaling—especially involving pyorubrin—may play a pivotal role in biofilm regulation. Molecular analysis also confirmed that *P. aeruginosa* Pr exhibited a markedly distinct behavior compared to both the *P. aeruginosa* ATCC 27853 and the other clinical strain *P. aeruginosa* Pc. In fact, the expression levels of the QS genes *lasI, lasR, rhlI*, and *rhlR* remained essentially unchanged across all cultural conditions. This absence of transcriptional responsiveness suggests that the QS regulatory network in *P. aeruginosa* Pr may be uncoupled from environmental inputs—possibly due to constitutive gene expression, rewired or attenuated regulatory circuits, or mutations affecting upstream sensory components such as two-component systems or autoinducer synthesis pathways. Such a decoupling from environmental modulation may represent an adaptive strategy evolved in specific clinical contexts, where maintaining a stable QS output—regardless of external cues—confers a selective advantage. Conversely, it could also reflect a trade-off, limiting the strain’s capacity to dynamically respond to environmental stressors or compete in fluctuating niches. Altogether, these findings underscore the phenotypic and regulatory diversity among *P. aeruginosa* isolates, revealing that even core systems like QS can be differentially tuned across strains depending on their ecological or clinical origin.

Finally, the clinical strain *P. aeruginosa* Pc exhibited both commonalities and unique traits compared to *P. aeruginosa* ATCC 27853. Notably, *P. aeruginosa* Pc showed the highest overall growth among all tested strains, with significant enhancement specifically in LB -NaCl, contrasting with the laboratory strain’s comparable growth in LB and LB -NaCl. This suggests that *P. aeruginosa* Pc may have superior mechanisms for coping with osmotic or ionic stress, possibly through more efficient ion homeostasis. Like *P. aeruginosa* ATCC 27853, *P. aeruginosa* Pc increased pyoverdine production significantly in LB -YE/+Gln, reinforcing the role of glutamine and nutrient limitation in inducing siderophore synthesis. Pyocyanin production was also elevated in LB -NaCl, accompanied by a striking color change indicative of accumulated reduced pyocyanin, likely reflecting oxygen depletion due to rapid growth. Biofilm formation closely paralleled phenazine levels, with the strongest biofilms formed in LB -NaCl. *P. aeruginosa* Pc’s pronounced growth and metabolite production under salt depletion suggest adaptations that enable it to exploit environmental stressors to enhance virulence-associated traits more effectively than the laboratory strain. A comparable pattern was also observed in *P. aeruginosa* Pc strain. In this isolate, all four QS genes—*lasI*, *lasR*, *rhlI*, and *rhlR*—were upregulated in the NaCl-depleted LB medium relative to both standard LB and the glutamine-supplemented condition (in which yeast extract was replaced). This supports the notion that the transcriptional activation of QS genes in response to ionic stress is a conserved feature among different P. aeruginosa strains. However, the *P. aeruginosa* Pc strain may possess enhanced sensitivity to ionic fluctuations or an intrinsically more active QS regulatory network under stress conditions. These differences may reflect strain-specific regulatory tuning, enabling *P. aeruginosa* Pc to more efficiently integrate environmental signals into virulence-related outputs such as biofilm formation and phenazine production. Such molecular adaptations could contribute to the observed increase in fitness traits under NaCl-depleted conditions, including accelerated growth, elevated metabolite synthesis, and robust biofilm development. Overall, the *P. aeruginosa* Pc strain appears to exploit osmotic stress as a cue to enhance persistence and pathogenic potential more effectively than the laboratory strain. Overall, although growth dynamics are largely maintained among the three strains, metabolite production and biofilm formation show significant differences. The laboratory strain *P. aeruginosa* ATCC27853 is optimized for pyoverdine synthesis, especially under glutamine supplementation, consistent with its laboratory-adapted phenotype, and forms biofilms rapidly. In contrast, the clinical strains produce less pyoverdine but higher levels of phenazines (*P. aeruginosa* Pc produces pyocyanin and *P. aeruginosa* Pr produces pyorubrin), indicative of increased virulence potential. Phenazine production is stimulated by NaCl depletion, reflecting stress responses relevant to infection niches, and repressed by glutamine, highlighting nutrient-dependent regulation. Biofilm formation varies among clinical strains: *P. aeruginosa* Pc responds strongly under salt-depleted conditions, favoring persistence under stress, whereas *P. aeruginosa* Pr generally exhibits weaker biofilms, particularly in the presence of glutamine. Comparing the laboratory strain with genetically unrelated clinical isolates allows validation under standard conditions and distinction of conserved physiological traits from strain-specific characteristics reflecting adaptive strategies in environmental or clinical contexts.

## 5. Conclusions

These findings collectively highlight the complex and dynamic adaptability of *P. aeruginosa*, demonstrating that strains within the same species—but with different evolutionary histories—have developed distinct strategies to cope with environmental challenges. While *P. aeruginosa* ATCC 27853 serves as a valuable laboratory model exhibiting core regulatory responses to nutrient limitation and osmotic stress, clinical isolates such as *P. aeruginosa* Pr and *P. aeruginosa* Pc show unique metabolic, phenotypic, and regulatory adaptations that reflect their specific ecological niches and selective pressures.

Analysis of key quorum-sensing (QS) genes—*lasI*, *lasR*, *rhlI*, and *rhlR*—was evaluated in biofilms grown under different LB-based culture conditions. In both *P. aeruginosa* ATCC 27853 and *P. aeruginosa* Pc, these genes were significantly upregulated in LB medium lacking NaCl, compared to standard LB and to LB where yeast extract was replaced with glutamine. This indicates that ionic stress, rather than nutrient substitution, strongly enhances QS activation. Notably, *P. aeruginosa* Pc exhibited even higher expression levels than the laboratory strain, suggesting a heightened sensitivity and responsiveness to NaCl depletion. In contrast, *P. aeruginosa* Pr strain showed no significant variation in QS gene expression across all tested conditions, implying a lack of environmental responsiveness or potentially constitutive regulation of its QS system. These results underscore how evolutionary divergence shapes the balance between survival, virulence, and colonization strategies in *P. aeruginosa* strains. The stable QS profile of *P. aeruginosa* Pr, along with its differential regulation of phenazines and biofilm formation, contrasts with the enhanced metabolic output and stress responsiveness of *P. aeruginosa* Pc, highlighting distinct adaptive strategies within the species. This diversity in QS regulation and other adaptive mechanisms emphasizes the importance of studying multiple strains to capture the full spectrum of *P. aeruginosa*’s physiological plasticity and pathogenic potential.

However, the present study is limited by the relatively small number of strains analyzed and by the use of binary conditions for NaCl and glutamine, which may not fully represent the species’ heterogeneity or capture the complexity of environmental influences. Nonetheless, this approach allowed us to clearly identify the impact of NaCl and glutamine on key pathogenic traits, including growth, biofilm formation, secondary metabolite production, and quorum-sensing. Additionally, variations in cultivation parameters such as oxygenation, culture volume, temperature, and pH—factors known to influence bacterial metabolism and behavior—were not exhaustively controlled or systematically varied, and could further modulate adaptive responses. Future investigations incorporating a broader strain collection and addressing these environmental variables in a controlled manner will be crucial to deepen our understanding of how *P. aeruginosa* tailors its strategies to diverse and fluctuating conditions.

## Figures and Tables

**Table 1:** MIC values (milligrams per liter) of tested antimicrobial agents and relative interpretation ^1^ based on EUCAST Clinical Breakpoint ^2^.

ANTIBIOTICS	*P. aeruginosa* ATCC 27853		*P. aeruginosa Pr* (Pyorubrin Producer)		*P. aeruginosa Pc* (Pyocyanin Producer)	
	MIC	Interpretation	MIC	Interpretation	MIC	Interpretation
**Amikacin (AMK)**	4	S	2	S	4	S
**Cefepime (FEP)**	0.5	I	8	I	16	R
**Ceftazidime (CAZ)**	2	I	2	I	32	R
**Ceftazidime/Avibactam (CAZ/AVI)**	2	S	8	S	2	S
**Ceftazidime/Tazobactam (CAZ/TZP)**	1	S	1	S	1	S
**Ciprofloxacin (CIP)**	0.5	I	0.5	I	0.12	I
**Colistin (COL)**	2	S	2	S	≤0.5	S
**Imipenem (IPM)**	2	I	>8	R	2	I
**Meropem (MEM)**	0.5	S	>8	R	0.5	S
**Piperacillina/Tazobactam (PIP/TZP)**	≤4	I	32	R	>64	R

^1^ S: Susceptible; I: Intermediate; R: Resistant. ^2^ EUCAST Clinical Breakpoint (milligrams per liter): AMK S ≤ 16 and R > 16; FEP S ≤ 0.001 and R > 8; CAZ S ≤ 0.001 and R > 8; CAZ/AVI S ≤ 8 and R > 8; CAZ/TZP S ≤ 4 and R > 4; CIP CAZ S ≤ 0.001and R > 5; COL S ≤ 4 and R > 4; IPM S ≤ 0.001 and R > 4; MEM, S ≤ 2 and R > 8; TZP, S ≤ 0.001 and R > 16.

**Table 2:** Comparative table of cell viability and production of pyoverdine, pyocyanin, and biofilm by the three *P. aeruginosa* strains under different culture conditions.

**Cell Viability** **(CFU/mL)**	**Time (h)**	**Cultural** **Condition**	***P. aeruginosa*** **ATCC27853**	***P. aeruginosa* Pr**	***P. aeruginosa* Pc**
	24	CTR (LB)	6.4 × 10^9^ ± 3	8.3 × 10^9^ ± 0.9	1.3 × 10^10^ ± 0.7
	48	CTR (LB)	6.5 × 10^9^ ± 2.9	5.6 × 10^9^ ± 1	6.3 × 10^9^ ± 2.7
	72	CTR (LB)	2.4 × 10^9^ ± 1.1	2.7 × 10^9^ ± 0.8	2.5 × 10^9^ ± 1
	24	LB -NaCl	6.3 × 10^9^ ± 2.7	8.2 × 10^9^ ± 0.7	1.3 × 10^10^ ± 0.7
	48	LB -NaCl	2.4 × 10^9^ ± 0.3	2.7 × 10^9^ ± 1.2	2.5 × 10^9^ ± 1.1
	72	LB -NaCl	6.4 × 10^9^ ± 1.2	5.8 × 10^9^ ± 0.7	6.3 × 10^9^ ± 2.8
	24	LB -YE/+Gln	6.5 × 10^9^ ± 1.1	8.5 × 10^9^ ± 0.8	1.2 × 10^10^ ± 1.1
	48	LB -YE/+Gln	6.4 × 10^9^ ± 2.7	5.6 × 10^9^ ± 1.1	6.5 × 10^9^ ± 2.9
	72	LB -YE/+Gln	2.5 × 10^9^ ± 1.1	2.6 × 10^9^ ± 0.8	2.5 × 10^9^ ± 1.1
**Pyoverdine** **(485 nm/535 nm)**	**Time (h)**	**Cultural** **condition**	***P. aeruginosa*** **ATCC27853**	***P. aeruginosa*** **Pr**	***P. aeruginosa*** **Pc**
	48 h	CTR (LB)	4.60 × 10^3^ ± 0.16	1.42 × 10^3^ ± 0.38	2.13 × 10^3^ ± 0.18
	72 h	CTR (LB)	7.28 × 10^3^ ± 0.94	7.71 × 10^2^ ± 1.73	1.57 × 10^3^ ± 0.17
	48 h	LB -NaCl	7.28 × 10^3^ ± 2.23	7.71 × 10^2^ ± 4.57	1.57 × 10^3^ ± 0.6
	72 h	LB -NaCl	1.62 × 10^4^ ± 0.24	1.49 × 10^3^ ± 0.1	3.14 × 10^3^ ± 0.57
	48 h	LB -YE/+Gln	2.09 × 10^4^ ± 0.1	4.65 × 10^3^ ± 0.25	6.00 × 10^3^ ± 0.42
	72 h	LB -YE/+Gln	2.47 × 10^4^ ± 0.17	2.05 × 10^3^ ± 0.19	4.60 × 10^3^ ± 0.38
**Phenazine** **(OD_405_)**	**Time (h)**	**Cultural** **condition**	***P. aeruginosa*** **ATCC27853**	***P. aeruginosa*** **Pr**	***P. aeruginosa*** **Pc**
	48 h	CTR (LB)	0.33 ± 0.02	0.53 ± 0.04	0.39 ± 0.02
	72 h	CTR (LB)	0.32 ± 0.03	0.52 ± 0.02	0.38 ± 0.01
	48 h	LB -NaCl	0.32 ± 0.03	0.52 ± 0.05	0.38 ± 0.02
	72 h	LB -NaCl	0.53 ± 0.04	0.50 ± 0.02	0.53 ± 0.02
	48 h	LB -YE/+Gln	0.34 ± 0.02	0.38 ± 0.03	0.45 ± 0.03
	72 h	LB -YE/+Gln	0.30 ± 0.02	0.35 ± 0.05	0.43 ± 0.03
**Biofilm** **(OD_490_)**	**Time (h)**	**Cultural** **condition**	***P. aeruginosa*** **ATCC27853**	***P. aeruginosa*** **Pr**	***P. aeruginosa*** **Pc**
	48 h	CTR (LB)	0.60 ± 0.09	0.49 ± 0.02	0.44 ± 0.04
	72 h	CTR (LB)	0.47 ± 0.03	0.67 ± 0.06	0.54 ± 0.06
	48 h	LB -NaCl	0.47 ± 0.09	0.67 ± 0.02	0.54 ± 0.09
	72 h	LB -NaCl	0.73 ± 0.07	0.42 ± 0.04	0.78 ± 0.06
	48 h	LB -YE/+Gln	0.57 ± 0.08	0.31 ± 0.02	0.43 ± 0.04
	72 h	LB -YE/+Gln	0.55 ± 0.02	0.23 ± 0.03	0.44 ± 0.05

## Data Availability

The original contributions presented in this study are included in the article. Further inquiries can be directed to the corresponding author.

## Supplementary Materials

The following supporting information can be downloaded at: https://www.mdpi.com/article/10.3390/microorganisms13092198/s1, Table S1: Tukey’s multiple comparisons test for Cinetic Growth production from *P. aeruginosa* ATCC27853; Table S2: Tukey’s multiple comparisons test for Pyoverdine production from *P. aeruginosa* ATCC27853; Table S3: Tukey’s multiple comparisons test for Pyocyanin production from *P. aeruginosa* ATCC27853; Table S4: Tukey’s multiple comparisons test for Biofilm production from *P. aeruginosa* ATCC27853; Table S5: Tukey’s multiple comparisons test for key quorum-sensing genes from *P. aeruginosa* ATCC27853; Table S6: Tukey’s multiple comparisons test for Cinetic Growth production from *P. aeruginosa* Pr; Table S7: Tukey’s multiple comparisons test for Pyoverdine production from *P. aeruginosa* Pr; Table S8: Tukey’s multiple comparisons test for Pyorubrin production from *P. aeruginosa* Pr; Table S9: Tukey’s multiple comparisons test for Biofilm production from *P. aeruginosa* Pr; Table S10: Tukey’s multiple comparisons test for key quorum-sensing genes from *P. aeruginosa* Pr; Table S11: Tukey’s multiple comparisons test for Cinetic Growth production from *P. aeruginosa* Pc; Table S12: Tukey’s multiple comparisons test for Pyoverdine production from *P. aeruginosa* Pc; Table S13: Tukey’s multiple comparisons test for Pyocyanin production from *P. aeruginosa* Pc; Table S14: Tukey’s multiple comparisons test for Biofilm production from *P. aeruginosa* Pc; Table S15: Tukey’s multiple comparisons test for key quorum-sensing genes from *P. aeruginosa* Pc.
