# Supplementary material for: Role of NaCl and Glutamine on Biofilm Production from Pseudomonas aeruginosa
Source: Microorganisms. 2025 Sep 19;13(9):2198. doi: 10.3390/microorganisms13092198 (PMC12472604; doi:10.3390/microorganisms13092198)
Supplement: Supplementary file 1 [file microorganisms-13-02198-s001.zip › microorganisms-3801465-supplementary.pdf]

Article

Role of NaCl and Glutamine on Biofilm Production from *Pseudomonas aeruginosa*

Table S1: Tukey's multiple comparisons test for Cinetic Growth production from *P. aeruginosa* ATCC27853

Time	Cultural Conditions	Mean Diff.	95.00% CI of diff.	Significant?	Summary	Adjusted P Value
24h	CTR (LB) vs. LB -NaCl	-0,05000	-0,1022 to 0,002151	No	ns	0,0585
	CTR (LB) vs. LB -YE/+Gln	0,1700	0,1178 to 0,2222	Yes	***	0,0001
	LB -NaCl vs. LB -YE/+Gln	0,2200	0,1678 to 0,2722	Yes	***	<0,0001
48h	CTR (LB) vs. LB -NaCl	-0,04333	-0,09680 to 0,01014	No	ns	0,1038
	CTR (LB) vs. LB -YE/+Gln	0,1633	0,1099 to 0,2168	Yes	***	0,0002
	LB -NaCl vs. LB -YE/+Gln	0,2067	0,1532 to 0,2601	Yes	***	<0,0001
72h	CTR (LB) vs. LB -NaCl	-0,07333	-0,1560 to 0,009335	No	ns	0,0770
	CTR (LB) vs. LB -YE/+Gln	0,1200	0,03733 to 0,2027	Yes	*	0,0102
	LB -NaCl vs. LB -YE/+Gln	0,1933	0,1107 to 0,2760	Yes	**	0,0009

Table S2: Tukey's multiple comparisons test for Pyoverdine production from *P. aeruginosa* ATCC27853

Time	Cultural Conditions	Mean Diff.	95.00% CI of diff.	Significant?	Summary	Adjusted P Value
48h	CTR (LB) vs. LB -NaCl	-11572	-15111 to -8034	Yes	***	0,0001
	CTR (LB) vs. LB -YE/+Gln	-16300	-19838 to -12762	Yes	***	<0,0001
	LB -NaCl vs. LB -YE/+Gln	-4728	-8266 to -1189	Yes	*	0,0150
72h	CTR (LB) vs. LB -NaCl	-12510	-17050 to -7971	Yes	***	0,0004
	CTR (LB) vs. LB -YE/+Gln	-17427	-21966 to -12887	Yes	***	<0,0001
	LB -NaCl vs. LB -YE/+Gln	-4916	-9456 to -377,1	Yes	*	0,0367

Table S3: Tukey's multiple comparisons test for Pyocyanin production from *P. aeruginosa* ATCC27853

Time	Cultural Conditions	Mean Diff.	95.00% CI of diff.	Significant?	Summary	Adjusted P Value
48h	CTR (LB) vs. LB -NaCl	-0,2033	-0,2600 to -0,1467	Yes	***	<0,0001
	CTR (LB) vs. LB -YE/+Gln	-0,01333	-0,06997 to 0,04330	No	ns	0,7601
	LB -NaCl vs. LB -YE/+Gln	0,1900	0,1334 to 0,2466	Yes	***	0,0001
72h	CTR (LB) vs. LB -NaCl	-0,1667	-0,2414 to -0,09197	Yes	**	0,0012
	CTR (LB) vs. LB -YE/+Gln	0,02000	-0,05469 to 0,09469	No	ns	0,7045
	LB -NaCl vs. LB -YE/+Gln	0,1867	0,1120 to 0,2614	Yes	***	0,0006

Table S4: Tukey's multiple comparisons test for Biofilm production from *P. aeruginosa* ATCC27853

Time	Cultural Conditions	Mean Diff.	95.00% CI of diff.	Significant?	Summary	Adjusted P Value
48h	CTR (LB) vs. LB -NaCl	-0,1300	-0,3466 to 0,08664	No	ns	0,2354
	CTR (LB) vs. LB -YE/+Gln	0,02667	-0,1900 to 0,2433	No	ns	0,9253
	LB -NaCl vs. LB -YE/+Gln	0,1567	-0,05997 to 0,3733	No	ns	0,1461
72h	CTR (LB) vs. LB -NaCl	-0,3700	-0,4769 to -0,2631	Yes	***	0,0001

CTR (LB) vs. LB -YE/+Gln	-0,08333	-0,1903 to 0,02361	No	ns	0,1173
LB -NaCl vs. LB -YE/+Gln	0,2867	0,1797 to 0,3936	Yes	***	0,0004

Table S5: Tukey's multiple comparisons test for key quorum sensing genes from *P. aeruginosa* ATCC27853

GENE	Cultural Conditions	Mean Diff.	95.00% CI of diff.	Significant?	Summary	Adjusted P Value
lasR	CTR (LB) vs. LB -NaCl	-0,3097	-0,4671 to -0,1522	Yes	**	0,0023
	CTR (LB) vs. LB -YE/+Gln	-0,06933	-0,2268 to 0,08814	No	ns	0,4211
	LB -NaCl vs. LB -YE/+Gln	0,2403	0,08286 to 0,3978	Yes	**	0,0081
lasI	CTR (LB) vs. LB -NaCl	-0,2337	-0,4198 to -0,04753	Yes	*	0,0198
	CTR (LB) vs. LB -YE/+Gln	0,002000	-0,1841 to 0,1881	No	ns	0,9994
	LB -NaCl vs. LB -YE/+Gln	0,2357	0,04953 to 0,4218	Yes	*	0,0190
rhlR	CTR (LB) vs. LB -NaCl	-0,3777	-0,5192 to -0,2361	Yes	***	0,0004
	CTR (LB) vs. LB -YE/+Gln	-0,04900	-0,1906 to 0,09258	No	Ns	0,5691
	LB -NaCl vs. LB -YE/+Gln	0,3287	0,1871 to 0,4702	Yes	***	0,0009
rhlI	CTR (LB) vs. LB -NaCl	-0,3017	-0,5134 to -0,08994	Yes	*	0,0112
	CTR (LB) vs. LB -YE/+Gln	-0,05400	-0,2657 to 0,1577	No	ns	0,7265
	LB -NaCl vs. LB -YE/+Gln	0,2477	0,03594 to 0,4594	Yes	*	0,0268

Table S6: Tukey's multiple comparisons test for Cinetic Growth production from *P. aeruginosa* Pr

Time	Cultural Conditions	Mean Diff.	95.00% CI of diff.	Significant?	Summary	Adjusted P Value
24h	CTR (LB) vs. LB -NaCl	-0,04000	-0,09964 to 0,01964	No	ns	0,1793
	CTR (LB) vs. LB -YE/+Gln	0,03000	-0,02964 to 0,08964	No	ns	0,3378
	LB -NaCl vs. LB -YE/+Gln	0,07000	0,01036 to 0,1296	Yes	*	0,0264
48h	CTR (LB) vs. LB -NaCl	-0,02000	-0,09608 to 0,05608	No	ns	0,7130
	CTR (LB) vs. LB -YE/+Gln	0,05667	-0,01941 to 0,1327	No	ns	0,1342
	LB -NaCl vs. LB -YE/+Gln	0,07667	0,0005874 to 0,1527	Yes	*	0,0486
72h	CTR (LB) vs. LB -NaCl	-0,01667	-0,07746 to 0,04413	No	ns	0,6934
	CTR (LB) vs. LB -YE/+Gln	0,04667	-0,01413 to 0,1075	No	ns	0,1228
	LB -NaCl vs. LB -YE/+Gln	0,06333	0,002539 to 0,1241	Yes	*	0,0427

Table S7: Tukey's multiple comparisons test for Pyoverdine production from *P. aeruginosa* Pr

Time	Cultural Conditions	Mean Diff.	95.00% CI of diff.	Significant?	Summary	Adjusted P Value
48h	CTR (LB) vs. LB -NaCl	-65,62	-995,3 to 864,1	No	ns	0,9746
	CTR (LB) vs. LB -YE/+Gln	-3229	-4158 to -2299	Yes	***	<0,0001
	LB -NaCl vs. LB -YE/+Gln	-3163	-4093 to -2233	Yes	***	0,0001
72h	CTR (LB) vs. LB -NaCl	-123,3	-518,4 to 271,7	No	ns	0,6271
	CTR (LB) vs. LB -YE/+Gln	-1281	-1676 to -885,6	Yes	***	0,0001
	LB -NaCl vs. LB -YE/+Gln	-1157	-1552 to -762,3	Yes	***	0,0003

Table S8: Tukey's multiple comparisons test for Pyorubrin production from *P. aeruginosa* Pr

Time	Cultural Conditions	Mean Diff.	95.00% CI of diff.	Significant?	Summary	Adjusted P Value
48h	CTR (LB) vs. LB -NaCl	0,03333	-0,06653 to 0,1332	No	ns	0,5900
	CTR (LB) vs. LB -YE/+Gln	0,1500	0,05014 to 0,2499	Yes	**	0,0087
	LB -NaCl vs. LB -YE/+Gln	0,1167	0,01681 to 0,2165	Yes	*	0,0269
72h	CTR (LB) vs. LB -NaCl	0,06000	-0,01878 to 0,1388	No	ns	0,1257
	CTR (LB) vs. LB -YE/+Gln	0,1733	0,09455 to 0,2521	Yes	**	0,0013
	LB -NaCl vs. LB -YE/+Gln	0,1133	0,03455 to 0,1921	Yes	*	0,0107

Table S9: Tukey's multiple comparisons test for Biofilm production from *P. aeruginosa* Pr

Time	Cultural Conditions	Mean Diff.	95.00% CI of diff.	Significant?	Summary	Adjusted P Value
48h	CTR (LB) vs. LB -NaCl	0,06333	0,01323 to 0,1134	Yes	*	0,0192
	CTR (LB) vs. LB -YE/+Gln	0,1733	0,1232 to 0,2234	Yes	***	0,0001
	LB -NaCl vs. LB -YE/+Gln	0,1100	0,05990 to 0,1601	Yes	**	0,0013
72h	CTR (LB) vs. LB -NaCl	0,1900	0,08536 to 0,2946	Yes	**	0,0034
	CTR (LB) vs. LB -YE/+Gln	0,4433	0,3387 to 0,5480	Yes	***	<0,0001

	LB -NaCl vs. LB -YE/+Gln	0,2533	0,1487 to 0,3580	Yes	***	0,0007

Table S10: Tukey's multiple comparisons test for key quorum sensing genes from *P. aeruginosa* Pr

GENE	Cultural Conditions	Mean Diff.	95.00% CI of diff.	Significant?	Summary	Adjusted P Value
lasR	CTR (LB) vs. LB -NaCl	-0,1520	-0,5700 to 0,2660	No	ns	0,5398
	CTR (LB) vs. LB -YE/+Gln	-0,02233	-0,4403 to 0,3956	No	ns	0,9853
	LB -NaCl vs. LB -YE/+Gln	0,1297	-0,2883 to 0,5476	No	ns	0,6306
lasI	CTR (LB) vs. LB -NaCl	0,05567	-0,2827 to 0,3941	No	ns	0,8718
	CTR (LB) vs. LB -YE/+Gln	0,09300	-0,2454 to 0,4314	No	ns	0,6922
	LB -NaCl vs. LB -YE/+Gln	0,05567	-0,2827 to 0,3941	No	ns	0,8718
rhlR	CTR (LB) vs. LB -NaCl	-0,001333	-0,4020 to 0,3993	No	ns	>0,9999
	CTR (LB) vs. LB -YE/+Gln	0,08933	-0,3113 to 0,4900	No	ns	0,7809
	LB -NaCl vs. LB -YE/+Gln	0,09067	-0,3100 to 0,4913	No	ns	0,7754
rhlI	CTR (LB) vs. LB -NaCl	-0,08667	-0,4100 to 0,2367	No	ns	0,7041
	CTR (LB) vs. LB -YE/+Gln	0,009333	-0,3140 to 0,3327	No	ns	0,9957
	LB -NaCl vs. LB -YE/+Gln	0,09600	-0,2274 to 0,4194	No	ns	0,6538

Table S11: Tukey's multiple comparisons test for Cinetic Growth production from *P. aeruginosa* Pc

Time	Cultural Conditions	Mean Diff.	95.00% CI of diff.	Significant?	Summary	Adjusted P Value
24h	CTR (LB) vs. LB -NaCl	-0,1600	-0,2219 to -0,09807	Yes	**	0,0005
	CTR (LB) vs. LB -YE/+Gln	0,07667	0,01474 to 0,1386	Yes	*	0,0210
	LB -NaCl vs. LB -YE/+Gln	0,2367	0,1747 to 0,2986	Yes	***	<0,0001
48h	CTR (LB) vs. LB -NaCl	-0,07333	-0,1167 to -0,02994	Yes	**	0,0049
	CTR (LB) vs. LB -YE/+Gln	0,03667	-0,006725 to 0,08006	No	ns	0,0907
	LB -NaCl vs. LB -YE/+Gln	0,1100	0,06661 to 0,1534	Yes	***	0,0006
72h	CTR (LB) vs. LB -NaCl	-0,06333	-0,1016 to -0,02507	Yes	**	0,0054
	CTR (LB) vs. LB -YE/+Gln	0,04000	0,001732 to 0,07827	Yes	*	0,0422
	LB -NaCl vs. LB -YE/+Gln	0,1033	0,06507 to 0,1416	Yes	***	0,0004

Table S12: Tukey's multiple comparisons test for Pyoverdine production from *P. aeruginosa* Pc

Time	Cultural Conditions	Mean Diff.	95.00% CI of diff.	Significant?	Summary	Adjusted P Value
48h	CTR (LB) vs. LB -NaCl	-1010	-2101 to 81,69	No	ns	0,0665
	CTR (LB) vs. LB -YE/+Gln	-3865	-4956 to -2773	Yes	***	<0,0001
	LB -NaCl vs. LB -YE/+Gln	-2855	-3946 to -1763	Yes	**	0,0005
72h	CTR (LB) vs. LB -NaCl	-889,8	-1908 to 128,6	No	ns	0,0811
	CTR (LB) vs. LB -YE/+Gln	-3032	-4051 to -2014	Yes	***	0,0002
	LB -NaCl vs. LB -YE/+Gln	-2142	-3161 to -1124	Yes	**	0,0016

Table S13: Tukey's multiple comparisons test for Pyocyanin production from *P. aeruginosa* Pc

Time	Cultural Conditions	Mean Diff.	95.00% CI of diff.	Significant?	Summary	Adjusted P Value
48h	CTR (LB) vs. LB -NaCl	-0,1400	-0,1935 to -0,08653	Yes	***	0,0005
	CTR (LB) vs. LB -YE/+Gln	-0,06333	-0,1168 to -0,009862	Yes	*	0,0254
	LB -NaCl vs. LB -YE/+Gln	0,07667	0,02320 to 0,1301	Yes	*	0,0108
72h	CTR (LB) vs. LB -NaCl	-0,1467	-0,1939 to -0,09943	Yes	***	0,0002
	CTR (LB) vs. LB -YE/+Gln	-0,05000	-0,09724 to -0,002761	Yes	*	0,0402
	LB -NaCl vs. LB -YE/+Gln	0,09667	0,04943 to 0,1439	Yes	**	0,0018

Table S14: Tukey's multiple comparisons test for Biofilm production from *P. aeruginosa* Pc

Time	Cultural Conditions	Mean Diff.	95.00% CI of diff.	Significant?	Summary	Adjusted P Value
48h	CTR (LB) vs. LB -NaCl	-0,3433	-0,4913 to -0,1954	Yes	***	0,0009
	CTR (LB) vs. LB -YE/+Gln	0,006667	-0,1413 to 0,1546	No	ns	0,9895
	LB -NaCl vs. LB -YE/+Gln	0,3500	0,2020 to 0,4980	Yes	***	0,0008
72h	CTR (LB) vs. LB -NaCl	-0,2767	-0,4129 to -0,1405	Yes	**	0,0019

CTR (LB) vs. LB -YE/+Gln	0,09667	-0,03953 to 0,2329	No	ns	0,1540
LB -NaCl vs. LB -YE/+Gln	0,3733	0,2371 to 0,5095	Yes	***	0,0004

Table S15: Tukey's multiple comparisons test for key quorum sensing genes from *P. aeruginosa* Pc

GENE	Cultural Conditions	Mean Diff.	95.00% CI of diff.	Significant?	Summary	Adjusted P Value
lasR	CTR (LB) vs. LB -NaCl	-0,7307	-1,072 to -0,3894	Yes	**	0,0015
	CTR (LB) vs. LB -YE/+Gln	-0,05967	-0,4009 to 0,2816	No	ns	0,8568
	LB -NaCl vs. LB -YE/+Gln	0,6710	0,3298 to 1,012	Yes	**	0,0023
lasI	CTR (LB) vs. LB -NaCl	-0,7147	-1,084 to -0,3456	Yes	**	0,0025
	CTR (LB) vs. LB -YE/+Gln	-0,02433	-0,3934 to 0,3447	No	ns	0,9778
	LB -NaCl vs. LB -YE/+Gln	0,6903	0,3213 to 1,059	Yes	**	0,0029
rhlR	CTR (LB) vs. LB -NaCl	-0,3957	-0,6388 to -0,1526	Yes	**	0,0059
	CTR (LB) vs. LB -YE/+Gln	0,07667	-0,1664 to 0,3198	No	ns	0,6216
	LB -NaCl vs. LB -YE/+Gln	0,4723	0,2292 to 0,7154	Yes	**	0,0024
rhlI	CTR (LB) vs. LB -NaCl	-0,2813	-0,4249 to -0,1378	Yes	**	0,0023
	CTR (LB) vs. LB -YE/+Gln	0,02833	-0,1152 to 0,1719	No	ns	0,8225
	LB -NaCl vs. LB -YE/+Gln	0,3097	0,1661 to 0,4532	Yes	**	0,0014
